# Obesity and Pancreatic Cancer: Insight into Mechanisms

**DOI:** 10.3390/cancers13205067

**Published:** 2021-10-10

**Authors:** Guido Eibl, Enrique Rozengurt

**Affiliations:** 1 Department of Surgery, David Geffen School of Medicine at UCLA, Los Angeles, CA 90095, USA; 2 Department of Medicine, David Geffen School of Medicine at UCLA, Los Angeles, CA 90095, USA; ERozengurt@mednet.ucla.edu

**Keywords:** obesity, pancreatic cancer, adipose tissue, intrapancreatic fat, inflammation, adipokine, gut microbiome

## Abstract

**Simple Summary:**

Obesity is recognized as a chronic progressive disease and risk factor for many human diseases. The high and increasing number of obese people may underlie the expected increase in pancreatic cancer cases in the United States. There are several pathways discussed that link obesity with pancreatic cancer. Adipose tissue and adipose tissue-released factors may thereby play an important role. This review discusses selected mechanisms that may accelerate pancreatic cancer development in obesity.

**Abstract:**

The prevalence of obesity in adults and children has dramatically increased over the past decades. Obesity has been declared a chronic progressive disease and is a risk factor for a number of metabolic, inflammatory, and neoplastic diseases. There is clear epidemiologic and preclinical evidence that obesity is a risk factor for pancreatic cancer. Among various potential mechanisms linking obesity with pancreatic cancer, the adipose tissue and obesity-associated adipose tissue inflammation play a central role. The current review discusses selected topics and mechanisms that attracted recent interest and that may underlie the promoting effects of obesity in pancreatic cancer. These topics include the impact of obesity on KRAS activity, the role of visceral adipose tissue, intrapancreatic fat, adipose tissue inflammation, and adipokines on pancreatic cancer development. Current research on lipocalin-2, fibroblast growth factor 21, and Wnt5a is discussed. Furthermore, the significance of obesity-associated insulin resistance with hyperinsulinemia and obesity-induced gut dysbiosis with metabolic endotoxemia is reviewed. Given the central role that is occupied by the adipose tissue in obesity-promoted pancreatic cancer development, preventive and interceptive strategies should be aimed at attenuating obesity-associated adipose tissue inflammation and/or at targeting specific molecules that mechanistically link adipose tissue with pancreatic cancer in obese patients.

## 1. Introduction

The prevalence of obesity in adults and children is increasing in the United States of America and in many other countries as well. Obesity is a well-recognized risk factor for a plethora of diseases, including pancreatic cancer. There are several potential mechanisms by which obesity may lead to an increase in pancreatic cancer incidence. However, the precise causal mechanism(s) are still poorly understood and may consist of a combination of local and systemic perturbations induced by the obese state. A better understanding of molecules and molecular signaling pathways driving pancreas cancer development and growth are of utmost importance to develop targeted prevention/interception strategies. The scope of the present review is not to provide a comprehensive, all-encompassing summary of all studies published on obesity and pancreatic cancer. Rather, the authors discuss selected mechanisms that in their opinion are of current interest and warrant further investigation.

## 2. Epidemiology of Obesity and Pancreatic Cancer

The global prevalence of obesity has almost tripled since 1975 [1]. In 2016, more than 1.9 billion adults (39%) were overweight (body mass index (BMI) ≥ 25) with over 650 million (13%) of those being obese (BMI ≥ 30) [1]. Alarmingly, more than 340 million children and adolescents (ages 5–19) were overweight or obese in 2016 and 39 million children younger than 5 years of age were overweight or obese in 2020 [1]. In the United States of America, the latest data brief from the National Center for Health Statistics reports a prevalence of obesity in adults aged 20 years or older of 42.4% in 2017–2018, which is a considerable increase from 33.7% in 2007–2008 [2]. There is no doubt that obesity is an enormous burden on the individual’s health and on society as a whole. Obesity itself is now declared a chronic progressive disease and in addition a risk factor for multiple human diseases including several types of cancer [3,4,5].

Pancreatic ductal adenocarcinoma (PDAC), the most common histologic subtype of cancers of the pancreas, continues to be a very aggressive and lethal type of cancer. The American Cancer Society has reported an estimated 60,430 new cases (28,480 females and 31,950 males) of pancreatic cancers in the year 2021 [6]. In the same calendar year, an estimated 22,950 female and 25,270 male patients will die of this disease, ranking pancreatic cancer as the third leading cause of cancer mortality in women and men combined [6]. In contrast, the estimates for pancreatic cancer prevalence in 2016 were 53,070 (25,400 females and 27,670 males) [7], which calculates to an almost 13.9% increase (12.1% in females and 15.5% in males) in total estimated pancreatic cancer cases over the last 5 years. Indeed, the mortality of pancreatic cancers is projected to surpass the deaths from colorectal cancer by 2030, catapulting pancreatic cancers to the second leading cause of cancer-related deaths in the United States [8]. The rising prevalence of obesity over the past decades may thereby be a significant contributing factor to the observed and expected increase in pancreatic cancer cases and mortality. 

A positive correlation between obesity and PDAC risk has been firmly established [3,9,10,11,12,13,14]. According to the National Institutes of Health 16.9% of all cases of PDAC in the United States can be attributed to obesity (in contrast cigarette smoking is estimated to be the causative factor in 10.2% of PDAC cases) [15]. In that context, the age of onset and duration of obesity seems to be an important factor in conferring PDAC risk. The length of time of being overweight was longer in patients with PDAC compared to controls, and the highest odds ratio for obesity was found in 30–39 year old subjects without diabetes [16]. Importantly, the association between obesity and risk of PDAC was stronger in men than in women [16]. Besides the importance of early adulthood, growing evidence suggests that adolescent and childhood obesity is also linked to an increased risk of developing PDAC later in life [17,18,19,20]. Here, it is imperative to distinguish the effect of obesity during early cancer development (risk factor for early tumor promotion) from its sometimes-paradoxical effects in the late-stage, advanced disease, where obesity occasionally appears to be associated with improved outcome (obesity paradox) [21,22,23]. It has been suggested that the lack of tumor cachexia (loss of muscle mass) in obese patients with advanced cancer may underlie the obesity paradox [24]. Taken together, the available evidence from epidemiologic studies clearly points to obesity as a risk factor for (early) PDAC development. Prevention of obesity, especially during childhood, adolescence, and early adulthood, is of paramount importance to curtail the expected rise in PDAC cases and mortality. A more detailed and comprehensive understanding of mechanisms that underlie the promotional effects of obesity on PDAC is necessary to identify and exploit potential molecular targets and to develop preventive and/or interceptive strategies. 

## 3. Mouse Models of Obesity and Pancreatic Cancer

It is widely accepted that *KRAS* mutations are critical initiating drivers of PDAC [25,26]. Preclinical animal models are instrumental for the study of risk factor-promoted PDAC development. Endogenous Kras models, which express mutated Kras conditionally from its endogenous gene locus, are considered as state-of-art models for PDAC and are widely used [27,28]. This model faithfully recapitulates human PDAC histopathologically and genetically, including the development of pancreatic intraepithelial neoplasias (PanIN-1 to -3), recognized precursor lesions of PDAC, and the presence of a desmoplastic reaction. For the conditional Kras mouse model of PDAC development *LSL-Kras^G12D^*, mice are normally crossed with either *ptf1a (p48)-Cre* or *pdx-Cre* mice (KC). Offspring with successful recombination will develop PanINs with complete penetrance [29]. About 5–10% of the animals will develop PDAC at about 9–12 months [29]. The KC mouse model has been crossed into various gene-deficient or mutated backgrounds, e.g., p53 (murine *Trp53*), most of which greatly accelerate the development of PDAC and shorten survival [30,31]. Since the transcription factors p48 or pdx are expressed in pancreatic progenitor cells during early pancreas development, all adult pancreas lineages, which include acinar, ductal, and endocrine lineages, harbor the mutated *Kras*. Studies have shown that KC mice do not accumulate additional changes in genes that are the most commonly mutated in human PDAC (e.g., *p16Ink4a*, *Trp53*, and *Smad4*) during PanIN development [32]. Besides the mouse model described above, in which oncogenic Kras is expressed during pancreatic embryological development, transgenic mouse models have been developed that allow for inducible and reversible expression of the oncogenic KrasG12D in the pancreas [33,34,35]. These inducible models are very valuable to study the temporal efficacy of oncogenic Kras to drive and maintain pancreatic carcinogenesis. Furthermore, using *elastase-cre* or *CK19-cre* (or *Sox9-cre*) strains, the expression of oncogenic Kras can be targeted to pancreatic acinar or ductal cells, respectively [36,37,38,39,40,41].

Preclinical studies employing the aforementioned mouse models have convincingly demonstrated that obesity accelerates PDAC development, thus providing an invaluable platform to study the obesity–PDAC link. Although oncogenic Kras mutations are thought of initiating factors for PDAC (see below), obesity is thought to be a tumor-promoting factor, especially during early neoplastic development. We and others have reported that (high-fat) diet-induced obesity (DIO) hastens the formation of PanINs and the progression to PDAC [37,42,43,44]. This is generally accompanied by weight gain and metabolic disturbances, e.g., hyperinsulinemia and hyperleptinemia, which are also seen in human obesity. In addition, DIO is accompanied by a strong fibro-inflammatory reaction in the pancreas of KC mice with elevated tissue levels of several pro-inflammatory cytokines, chemokines, and growth factors [42,43]. The importance of inflammation and the efficacy of anti-inflammatory drugs on PDAC development in KC mice has been reported previously [45]. Besides mouse models with DIO, a recent study showed that KC mice with genetic obesity (*Pdx1-Cre;LSL-KrasG12D/+* mice crossed with leptin-deficient [*ob/ob*] mice) also developed PDAC faster and succumbed to the disease earlier [46]. Taken together, there is unambiguous evidence from preclinical mouse models that obesity (DIO or genetic) promotes PDAC.

## 4. Mechanisms Linking Obesity and Pancreatic Cancer

Several mechanisms are usually discussed by which obesity may promote (pancreatic) cancer development and progression, including systemic chronic inflammation, adipokines, sex hormones, hyperinsulinemia, and gut microbiome [47,48,49,50,51]. Although certainly not exhaustive, selected potential mechanisms are discussed below.

### 4.1. Influence of Obesity on Kras Activity

Oncogenic mutations in *KRAS* are thought to be an initiating event in human PDAC, which is strongly supported by preclinical mouse models. It is well documented that the vast majority of human PDACs contain a *KRAS* mutation (most commonly *G12D*), which can be found also in early-stage PanINs [52]. Recent exome sequencing studies confirmed that *KRAS* is the most frequently mutated gene found in PDAC (~95%) [53,54]. However, data from mouse models have clearly shown that *Kras* mutant cells do not readily form early PanIN-1 lesions, despite the expression of mutant Kras in every pancreatic epithelial cell [29,55]. It seems that mutated *Kras* alone is rather insufficient to drive pancreatic neoplasia, but additional genetic, epigenetic, and/or microenvironmental changes may be required [55]. Non-genetic perturbations in the pancreatic microenvironment, as putatively induced in the obese state, may thereby be especially critical in the early stages of pancreatic neoplastic development [26,55]. 

Considering the importance of oncogenic *Kras* and the known promoting effects of obesity in PDAC, it is conceivable to consider a direct effect of obesity and/or the obesogenic diet on Kras activity. Based on the observation that only a small percentage of mutated Kras is occupied with guanosine triphosphate (GTP) and thus cannot be considered constitutively active [56], Logsdon and colleagues postulated a Ras/Inflammation Feed Forward model, in which oncogenic Kras requires activation by external/inflammatory factors, e.g., as found in obesity, to drive pancreatic neoplasia [57,58]. Once the activity of oncogenic Kras has exceeded a certain threshold, it can generate its own inflammatory mediators, which in turn feed-back to further stimulate Kras activity [57,58], thereby driving cancer development. These external/inflammatory mediators can comprise a variety of factors, including inflammatory cytokines, eicosanoids, and other ligands of receptor tyrosine kinases and G protein-coupled receptors (GPCRs) [57]. Based on this paradigm, the pancreas from *LSL-Kras/Ela-CreERT* mice, which were fed high-fat diets, had elevated Kras activity, elevated phospho ERK, increased pancreatic inflammation, fibrosis, and neoplastic lesions [37]. However, these considerations were based on pull down assays of Kras-GTP that do not take into account the effect of Kras tyrosine phosphorylation on the affinity for the Ras-binding domain of RAF [59]. In our opinion, it will be important to reinvestigate the activity of Kras in the setting of DIO using additional assays. In our own studies, we have observed activation of signaling molecules that may be downstream of Kras, e.g., increased levels of phosphorylated mitogen-activated protein kinase (MEK), extracellular signal-regulated kinases (ERK), and S6, in the pancreas of KC mice with DIO [60].

### 4.2. Adipose Tissue and Obesity-Associated Meta-Inflammation

Contrary to initial depictions as simply an energy storage tissue, adipose tissue (AT) is now known to be a metabolically and hormonally highly active and dynamic organ that is capable of responding to a variety of internal and external stimuli and synthesizing a large range of biologically active peptides. In turn, AT-derived mediators regulate many physiological and pathophysiological processes, e.g., food intake, insulin sensitivity, immunity, and inflammation [61,62]. These peptides include adipokines, e.g., leptin, adiponectin, and lipocalin-2, which are mainly secreted by adipocytes, as well as AT-derived factors, including IL-6 and TNF-α, which can be also be secreted by cells other than or in addition to adipocytes, e.g., macrophages [62]. Generally, AT can influence PDAC development systemically via soluble mediators that are released from distant (visceral) fat depots and reach the pancreatic microenvironment through the systemic circulation or via paracrine effects elicited by intrapancreatic adipocytes (see below) [63,64]. A mechanism that is receiving increasing attention is communication via extracellular vesicles from adipocytes that can fuse with target cells in the pancreas [65,66,67]. It has been shown recently that adipocytes experience strong energetic stress during obesity, which resulted in the release of small extracellular vesicles harboring respiration-competent, but oxidatively damaged, mitochondrial fragments, which access the systemic circulation and are internalized by cardiomyocytes [68]. It is intriguing to think that during obesity, adipocytes communicate with transformed and non-transformed pancreatic cells via extracellular vesicles, thereby promoting tumor development. Specific studies would be required to test this hypothesis.

#### 4.2.1. Visceral Adipose Tissue

The defining characteristic of obesity is the enlargement and expansion of white adipose tissue, which can occur via hyperplasia and/or hypertrophy. While adipocyte hyperplasia, which is more often seen in subcutaneous AT, is usually associated with low levels of AT inflammation and maintained insulin sensitivity, adipocyte hypertrophy predominates in visceral AT (VAT) and correlates with pro-inflammatory responses and impaired insulin sensitivity [69]. Hypertrophic adipocytes can experience hypoxia and undergo stress responses and cell death, leading to the secretion of pro-inflammatory factors, an increase in pro-inflammatory immune cell infiltration, and the deposition of lipid molecules ectopically in other organs. This is particularly important as the VAT (omental, mesenteric, and other intra-abdominal fat pads), and VAT inflammation is the main source of the systemic and local chronic inflammation in the obese state [70] and is more strongly associated with metabolic dysfunction and cancer than subcutaneous fat [12,71,72,73]. VAT (but not subcutaneous fat) is significantly correlated with the number of PanINs [74]. In our own studies, more PDACs developed in obese male KC mice [42], which displayed a preferential expansion of VAT, as compared to obese female mice that favored subcutaneous fat gain [75].

#### 4.2.2. Adipose Tissue Inflammation

**Figure 1:**
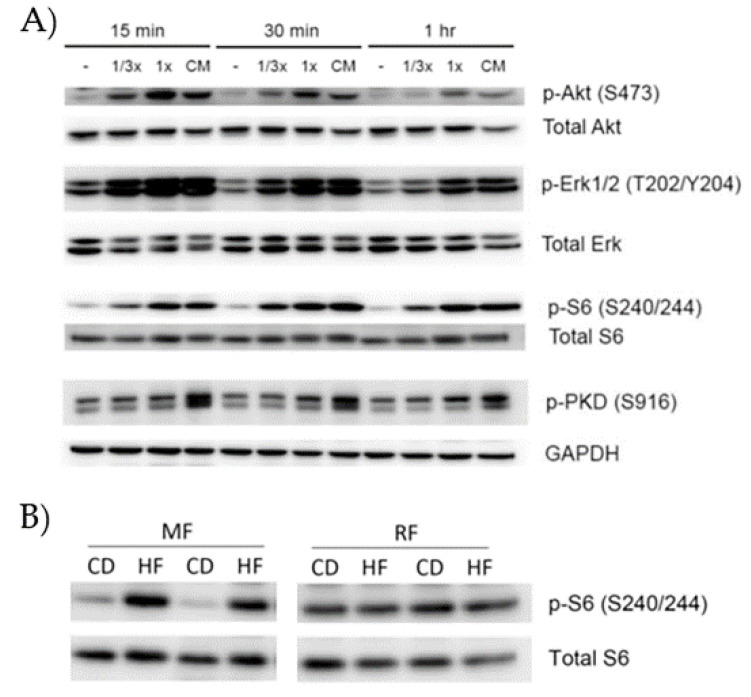
Soluble factors released from adipose tissue stimulate pro-oncogenic signaling pathways in pancreatic cancer cells. (**A**) Mesenteric adipose tissue from obese KC mice was harvested and cultured in vitro. Murine pancreatic cancer cells were incubated with the culture supernatant (undiluted (1×) or diluted (1/3×) for 15, 30, and 60 min and phosphorylation of pro-oncogenic signaling molecules detected by Western blotting. Total forms of the signaling molecules or glyceraldehyde 3-phosphate dehydrogenase (GAPDH) were used as loading controls. Serum-free culture medium (−) or complete culture medium with 10% fetal bovine serum (CM) served as negative and positive controls, respectively. (**B**) Mesenteric (MF) and retroperitoneal (RF) adipose tissues from obese (high fat: HF) or lean (control diet: CD) KC mice (*n* = 2 in each group) were harvested and cultured in vitro. Murine pancreatic cancer cells were incubated with the culture supernatant for 30 min and phosphorylation of S6 was measured by Western blotting. Total S6 served as a loading control. Data are authors’ own unpublished results.

VAT has been shown to be the predominant source of the systemic, low-grade, chronic inflammation seen in obesity (also called meta-inflammation [76]) and most important for the development of type 2 diabetes mellitus (T2DM) [77,78]. VAT contains a great variety of immune cells, the composition of which is significantly altered in the obese state [78]. An important early step in the development of obesity-induced VAT inflammation is the switch of anti-inflammatory M2 macrophages to a pro-inflammatory M1-like phenotype [78,79]. These M1-like macrophages are thought to be the main source of systemic pro-inflammatory cytokines in obese and/or diabetic subjects [78]. Obesity-associated AT inflammation is characterized by the generation and secretion of multiple inflammatory cytokines and chemokines, including but not limited to interleukin-6 (IL-6), tumor necrosis factor-α (TNF-α), IL-1β, IL-18, and monocyte chemoattractant protein-1 (MCP-1) [78]. It is conceivable that the systemic elevation of these inflammatory mediators, secreted from the VAT in obese subjects, may bind to their cognate receptors on transformed pancreatic epithelial cells, thereby conferring a proliferative and pro-survival benefit. Our studies (unpublished) showed that the cell culture supernatant of the VAT stromal vascular fraction (all cellular components except mature adipocytes) and of direct VAT explants of obese KC mice robustly stimulated DNA synthesis and oncogenic signaling pathways in murine PanIN cells (Figure 1). These results indicate that soluble factors from the VAT, in particular mesenteric fat, have direct proliferative effects on (pre)malignant pancreatic epithelial cells. 

Furthermore, the systemically elevated factors in the obese condition may also stimulate neoplastic pancreatic epithelial cells and/or non-malignant cells in the pancreatic stroma, e.g., cancer-associated fibroblasts, infiltrating/resident immune cells, and intrapancreatic adipocytes (see below), to generate locally inflammatory cytokines and chemokines. Locally produced chemokines can further recruit immune cells, e.g., circulating monocytes, into the pancreatic microenvironment, where they can contribute to and reinforce local tissue inflammation and cancer cell growth [80]. The importance of paracrine signaling in the pancreatic microenvironment to oncogenic Kras-driven metabolic reprogramming and tumor growth has recently been reported [81]. We previously demonstrated elevated pancreatic tissue levels of several cytokines and chemokines, e.g., IL-6, TNF-α, and MCP-1, in obese KC mice that were fed a diet high in fat and calories [43]. Interestingly, while lean KC mice (fed a control diet) also had elevated pancreatic tissue levels of these cytokines/chemokines compared to wild-type mice (lean and obese), obese KC had even significantly higher levels [43], suggesting a positive reinforcement between oncogenic Kras and obesity/high-fat diet.

#### 4.2.3. Leptin

The first adipokine identified in 1994, leptin, is a 16 kDa hormone encoded by the *ob* gene, the murine homologue of the human *LEP* gene [82]. It binds to leptin receptors (Ob-R), members of the class I cytokine receptor family, and activates mainly the Janus kinase/signal transducer and activator of transcription (JAK/STAT) signaling pathway [83,84,85]. Leptin is secreted mainly by adipocytes and its primary function is to decrease food intake and increase energy expenditure (anorexigenic) mainly through its actions in the hypothalamus [86,87]. As leptin expression correlates to adipose mass, obese subjects display elevated plasma leptin levels [88,89]. Since elevated leptin levels in obese patients do not lead to decreased food intake and increased energy expenditure, the obesity-associated hyperleptinemia is thought to reflect leptin resistance [90]. The role of leptin in PDAC development and growth is still controversial. In a pooled, nested case-control study, increased leptin concentrations correlated with pancreatic cancer, but only after a long follow-up of 10 or more years [91]. In another prospective, nested case-control study, higher leptin levels correlated with an increased risk of pancreatic cancer in men, but not women [92]. Results from a large Mendelian randomization study did not support a causal effect of plasma leptin levels on pancreatic cancer development [93]. In preclinical animal studies, KC mice with DIO developed hyperleptinemia [42,43], suggesting a relationship between leptin and PDAC. Caloric restriction in KC mice was associated with decreased leptin levels and a delay in PDAC development [94]. However, KC mice with genetic obesity (*Pdx1-Cre;LSL-KrasG12D/+* mice crossed with *ob/ob* mice) that are leptin deficient also developed PDAC faster, arguing against a causative role of leptin in PDAC [46,95]. Furthermore, KC mice with genetic deficiency in hormone-sensitive lipase had decreased plasma leptin levels but accelerated PDAC development [96]. In vitro studies have demonstrated that PDAC cells express functional leptin receptors (Ob-Rb) and exposure to leptin stimulates migration, invasion, and proliferation of PDAC cells [97,98,99]. These effects were mediated by activation of several signaling pathways, including the phosphoinositide 3-kinase/protein kinase B (PI3K/Akt) and JAK2/STAT3 pathways. In addition, the leptin–Notch axis seems to play a role in proliferation and chemoresistance in PDAC cell cultures [100,101,102]. Taken together, although in vitro experiments clearly demonstrated functional leptin receptor expression and various direct effects of leptin in PDAC cells, the cumulative data from preclinical animal and epidemiologic studies at least question a causative role of leptin in obesity-promoted PDAC.

#### 4.2.4. Adiponectin

First described in 1995 [103], adiponectin is a ~30 kDa protein secreted mostly by the white adipose tissue and possesses anti-diabetic, anti-inflammatory, anti-atherogenic, and anti-angiogenic properties [104,105]. Adiponectin binds to AdipoR1 and AdipoR2 receptors, which, contrary to all known G protein-coupled receptors (GPCRs), are seven transmembrane domains containing membrane proteins that have their amino terminus intracellularly and their carboxy terminus extracellularly [105]. Binding of adiponectin to its receptors leads to hydrolysis of ceramide to sphingosine. Sphingosine kinases can then phosphorylate sphingosine to sphingosine-1-phosphate (S1P) [104], which activates S1P receptors, a class of GPCRs. Further downstream of ceramide hydrolysis and sphingosine formation, but also through ceramide-independent pathways, AdipoR can activate PI3K, Akt, adenosine monophosphate-activated protein kinase (AMPK), and calcium (Ca^2+^) release [104,106]. Despite its secretion by adipocytes, adiponectin plasma levels are paradoxically decreased in obesity [104,107,108]. The currently available evidence of an association of adiponectin levels and PDAC risk is conflicting. Higher adiponectin concentrations were found to be inversely associated with PDAC in male smokers, which was significant among cases diagnosed 5 or more years after blood collection [109]. Analyzing 468 PDAC cases and 1080 matched controls from five prospective US cohorts, plasma adiponectin was inversely associated with PDAC risk [110]. A case-control study showed no association of adiponectin with PDAC risk overall; however, higher adiponectin levels were associated with a reduction in PDAC risk among never smokers [111]. An influence of smoking on the risk of adiponectin on PDAC risk has been reported [112]. However, a Mendelian randomization study did not find an association of adiponectin with PDAC risk [93]. Furthermore, other studies reported higher adiponectin levels in patients PDAC [113,114,115]. However, in these studies, adiponectin levels were measured at the time of diagnosis or treatment of PDAC and the true effect of adiponectin on PDAC risk may have been obscured. In preclinical murine studies, an AdipoR agonist (AdipoRon) decreased the growth of PDAC xenografts [116,117]. Conversely, deficiency or knockdown of adiponectin receptors markedly promoted PDAC xenograft growth [118,119]. Interestingly, a recent xenograft study showed that AdipoRon failed to suppress PDAC growth in mice with DIO, while it suppressed tumor growth in lean mice [120]. Taken together, a link between adiponectin and PDAC risk is not conclusively demonstrated by available epidemiologic studies. Preclinical evidence provides a stronger support of a role of adiponectin in PDAC therapy. Further, carefully designed studies are clearly needed to answer the question whether low adiponectin levels as seen in obese subjects causally increase the risk of developing PDAC. 

#### 4.2.5. Lipocalin-2

Another interesting molecule that may provide a link between obesity and PDAC is lipocalin-2 (LCN2). LCN2, also known as neutrophil gelatinase-associated lipocalin (NGAL), is a 25 kDa protein expressed and secreted by various cell types, which belongs to a large group of small extracellular proteins with a variety of biological functions, including iron homeostasis, inflammation, innate immunity, and energy metabolism [121,122,123,124]. LCN2 is also considered an adipokine. It is secreted from adipocytes and its production is highly regulated by metabolic stress, inflammatory cytokines, and nutrient signals [124]. Elevated LCN2 levels are typically found in obese humans and mouse models of obesity [125,126]. LCN2 has been implicated in the pathogenesis of various pancreatic diseases, including PDAC, acute and chronic pancreatitis, and T2DM [127]. LCN2 is upregulated in PDAC mouse models, correlates to decreased food intake, and its absence protects from tumor cachexia, presumably via a type 4 melanocortin receptor-mediated mechanism [128]. Increased LCN2 expression has been found in human PanINs and PDAC [129,130,131]. However, preclinical studies reported contradictory, pro- and anti-tumor functions of LCN2 in PDAC [132,133,134]. Using *Kras^G12D^;Ela-CreERT;Lcn2^−/−^* mice (whole body deletion of LCN2) Cruz-Monserrate and colleagues showed that lack of LCN2 prevents weight gain and obesity when fed a high-fat diet [135]. This was associated with reduced pancreatic fibro-inflammation, PanIN formation, and prolonged survival, indicating a tumor-promoting role of LCN2 in PDAC [135]. However, it is not clear whether the reduced tumor growth in *Kras^G12D^;Ela-CreERT;Lcn2^−/−^* mice was directly due to the lack of LCN2 effects on the pancreas or indirectly to the reduced weight gain (anti-obesity effects). Further orthotopic syngeneic studies showed that lack of LCN2 in the host significantly diminished pancreatic fibrosis and inflammation and attenuated growth of (LCN2 expressing) PDAC cells [135], pointing to the importance of paracrine LCN2 (rather than autocrine) effects in PDAC. Taken together, it is conceivable that elevated LCN2 during obesity is a driver of PDAC development and progression, although further studies are clearly needed to cement that hypothesis.

#### 4.2.6. FGF21

As a novel fibroblast growth factor (FGF) family member identified in 2000 [136], FGF21, has emerged as an important regulator and orchestrator of glucose and lipid metabolism and energy homeostasis [137,138] with the potential to treat obesity, T2DM, and meta-inflammation [139,140,141,142]. FGF21 is considered a “master sensitizer” of metabolic hormonal signals [143]. Circulating FGF21 levels are elevated in diabetic and obese subjects [144] and administration of FGF21 has been shown to enhance insulin sensitivity and reverses obesity by increasing energy expenditure [145,146]. On target cells, FGF21 binds to and activates a receptor complex of FGF receptor 1c (FGFR1c) and its co-receptor β-Klotho (KLB); however, little is known about the downstream intracellular signaling events [143]. Exciting recent studies implicate FGF21 in obesity-promoted PDAC [147,148,149]. Lu and colleagues reported that FGF21, its target receptor FGF receptor 1 (FGFR1), and its co-receptor β-Klotho (KLB) were expressed in normal pancreatic acinar cells and showed that FGF21 levels were decreased downstream of oncogenic Kras [147]. Administration of recombinant human FGF21 to *Kras^G12D^;Ela-CreERT* mice fed a high-fat diet attenuated pancreatic and systemic inflammation, suppressed PanIN formation and PDAC development, and prolonged the survival of *Kras^G12D/+^* mice [147]. The authors concluded that downregulation of pancreatic FGF21 by oncogenic Kras renders the pancreas vulnerable to an obesogenic high-fat diet, leading to enhanced inflammation and the development of PDAC. It is relevant that acute and chronic pancreatitis, a condition that increases PDAC incidence in preclinical models and human patients, has been also associated with a marked decrease of FGF21 in the pancreas [150].

#### 4.2.7. Wnt5a

Recent studies have demonstrated that the pro-inflammatory adipokine wingless-type mouse mammary tumor virus integration site family member 5A (Wnt5a), together with the anti-inflammatory secreted frizzled-related protein 5 (Sfrp5), which both signal via the non-canonical Wnt pathway, play a key role in the pathogenesis of obesity and its metabolic complications [151,152]. The circulating levels of Wnt5a and its expression in VAT are increased in obesity [152,153], contributing to systemic and local inflammation [154]. Furthermore, overexpression of Wnt5a has been reported in human PDAC [155] and has a profound impact on the PDAC microenvironment [156]. The link between adipose tissue, Wnt5a, and PDAC has gained enormous significance, as Wnt5a has been shown to positively correlate with Yes-associated protein (YAP) activity (see below) in human PDAC [157]. Wnt5a leads to YAP activation, which drives the aggressive squamous subtype of PDAC, which is YAP dependent but Kras independent [157]. We postulate that elevated levels of Wnt5a during obesity (released from the VAT) may promote the development and growth of the aggressive squamous subtype of PDAC through YAP-mediated mechanisms, although further studies are needed to confirm this model. 

#### 4.2.8. Intrapancreatic Fat

While the link between general and visceral adiposity to PDAC is well established, studies on the functional significance of intrapancreatic fat on pancreatic carcinogenesis and cancer promotion are very scarce and just recently have attracted increased attention. While the presence of fat in the pancreas has been first described almost a century ago [158] and thought of as a bystander of several underlying diseases, there is now increased recognition that intrapancreatic fat deposition, variably also called pancreatic steatosis or fatty pancreas disease, has a role in T2DM, pancreatitis, and pancreatic cancer [159,160,161,162]. While several histological studies have demonstrated a small amount of intrapancreatic fat in the majority of normal pancreata [163,164], excess intrapancreatic fat is now discussed to be important in the development of endocrine and exocrine pancreatic diseases. Excess intrapancreatic fat can originate from the formation and expansion of intra- and/or interlobular fat [165], which are not exclusive but can be present simultaneously in the same organ. Intracellular fat droplets (positive for adipose differentiation–related protein (ADFP)) have been found in pancreatic endocrine cells [166,167] and acinar cells after high-fat diets [168,169]. Intra- and interlobular perilipin-positive adipocytes have been detected in the human and mouse pancreas, which was increased in mice after a high-fat diet [80,169]. The source of these adipocytes may be mesenchymal stem cells in the pancreatic stroma, which can differentiate into adipocytes [170]. AT-derived stem or progenitor cells have been reported to be able to home to the tumor stroma promoting cancer progression [171,172]. During obesity, AT-derived progenitor cells, which are elevated and mobilized in obesity, may differentiate into adipocytes in the pancreatic microenvironment. The importance of the visceral mesothelium, which also covers the pancreas, in pancreatic cancer biology and as a source of adipocytes has been reported [173,174,175,176]. However, a recent study refutes mesothelial cells as a source of adipocytes in mice [177]. In addition, it has been reported that an acinar-to-adipocyte transdifferentiation program exists, possibly driven by inflammation [178]. Finally, pancreatic stellate cells have been shown in vitro to be able to transdifferentiate into adipocyte-like cells [179]. Our knowledge of the importance of intrapancreatic fat for the development and progression of pancreatic cancer is still in its infancy, and preclinical studies are necessary to illuminate its functional significance. It is conceivable that the formation and expansion of intrapancreatic fat (intra- and/or interlobular) that may be seen during obesity has a profound paracrine metabolic and proliferative effect on transformed pancreatic epithelial cells via local secretion of adipokines and other adipose-derived inflammatory cytokines. In addition, intrapancreatic adipocytes may also be an important source of free fatty acids, which can be used by malignant cells as fuel and for membrane synthesis [180] as well as paracrine signals.

### 4.3. Insulin and Insulin-Like Growth Factor-1

Patients with long-standing T2DM have an increased risk of PDAC [13,14,181,182]. In addition, T2DM is often associated with obesity, which by itself also promotes PDAC development (see above). Patients with T2DM and obesity are often characterized by long periods of elevated intrapancreatic insulin levels caused by the pancreatic β-cells trying to overcome the insulin resistance present in T2DM and obesity to maintain glucose homeostasis. Elevated levels of insulin, particularly intrapancreatic, can reach pancreatic acinar and ductal cells adjacent to pancreatic islets via the intrapancreatic portal circulation, activate insulin and IGF-1 receptors present on these cells, and may thus conceivably contribute to the PDAC growth [182]. The importance of insulin is underscored by mouse models of PDAC. For example, KC mice subjected to DIO consistently developed hyperinsulinemia and elevated levels of IGF-1 [42,43,44,94]. Furthermore, the anti-diabetic drug metformin significantly suppressed PDAC development in KC mice with DIO, which was associated with a normalization of the hyperinsulinemia [60]. In vitro experiments have clearly demonstrated the proliferative action of insulin on PDAC cells [183,184,185,186,187,188]. A crosstalk between insulin/IGF-1 receptors and G protein-coupled receptor signaling systems has been identified that converges on the mechanistic target of rapamycin (mTOR), which is inhibited by metformin [184,185,186,187,189]. Furthermore, the crosstalk between the insulin receptor and GPCRs has been shown to potently stimulate YAP through PI3K and protein kinase D in PDAC cells [183]. YAP and its paralogue transcriptional co-activator with PDZ-binding motif (TAZ), as major downstream effectors of the Hippo pathway, have recently gained enormous interest as critical molecules in PDAC formation and progression [157,190,191,192,193,194,195,196,197]. Interestingly, the expression of YAP and TAZ in the pancreas is increased in KC mice with DIO, which is downregulated by metformin [60]. Taken together, it has been conclusively shown that insulin receptor and GPCRs signaling pathways stimulate PDAC cell proliferation by converging on mTOR. In obesity, elevated levels of insulin/IGF-1 and gastrointestinal peptides that act via their cognate GPCRs, e.g., neurotensin [198], enhance the crosstalk between insulin receptor and GPCR signaling pathways, leading to increased cellular proliferation. Targeting this insulin receptor/GPCR crosstalk with the antidiabetic drug metformin may have potent beneficial effects on PDAC development [199]. In addition to the importance of hyperinsulinemia, elevated glucose levels and advanced glycation end products (AGE) may also play an important role. In this context, the receptor for AGEs (RAGE) has been shown to be a tumor promoting factor in PDAC and to orchestrate the interplay between metabolic diseases, inflammation, and cancer [200,201,202].

### 4.4. Gut Microbiome

Human studies and animal models have demonstrated that the gut microbiota is altered in obesity [203,204,205]. Generally, the microbial diversity as seen in healthy individuals is decreased in obese subjects. In particular, an abundance of *Firmicutes* (increase in the *Firmicutes:Bacteroidetes* ratio) was found in mice with diet-induced and genetic obesity [206,207,208]. Additionally, animal studies provide evidence that changes in the gut microflora are causally linked to the development of obesity and T2DM [205,209,210]. Furthermore, there is strong evidence that altered gut microbiota are critical for the development of colorectal cancer [211]. In another study, dietary or genetic obesity induced changes in the gut microbiota, which facilitated the development of hepatocellular carcinoma in mice through an increase of deoxycholic acid [212]. The gut microbiome has been implicated in PDAC as well [213,214,215]. Importantly, the presence and significance of an intrapancreatic, intratumoral microbiome that crosstalks with the gut microbiome has been reported [216,217,218]. In addition to the microbiome, the fungal mycobiome has recently been shown to promote pancreatic carcinogenesis [219]. Our own studies have shown that oral administration of metformin to KC mice fed an obesogenic high-fat diet normalized the high diet-induced gut dysbiosis [220]. We found that oral administration of metformin to obese KC mice lowered the abundance of the genus *Clostridium sensu stricto* and significantly increased the levels of *Akkermansia* [220]. *Akkermansia muciniphila*, an intestinal symbiotic bacterium, plays an important role in maintaining a functioning gut barrier [221,222,223]. Data from human studies confirmed that the abundance of *Akkermansia muciniphila* correlates to a lower incidence of obesity and other metabolic diseases [224,225]. 

Changes in the gut microbiome, caused by genetic, environmental, or nutritional factors, are thought to influence the development of metabolic diseases and cancer by several mechanisms. These include microbiota-derived metabolites, e.g., short-chain fatty acids (mainly acetate, propionate, and butyrate), activation of intestinal GPCRs, or translocation of bacteria or bacterial components, e.g., lipopolysaccharide (LPS), enabled by an increase in gut permeability, leading to a systemic pro-inflammatory state [205]. It is well known that obesity is associated with elevated circulating LPS levels (metabolic endotoxemia) [226,227,228]. Animal models showed that obesity-associated metabolic endotoxemia induced AT inflammation through an LPS/toll-like receptor 4 (TLR4)-mediated mechanism [207,228,229]. Microbial alterations in PDAC featured an increase of certain pathogens and LPS-producing bacteria [230]. Although, to our knowledge, no studies have been published that directly measured LPS levels in the pancreas of obese subjects with PDAC, other reports using obese rats with deficiency in TLR4 have implicated an important role of LPS for the pancreatic β-cell function in [231]. Furthermore, exogenous administration of LPS to mice with oncogenic Kras expression in pancreatic acinar cells led to chronic pancreatitis and neoplastic PanIN formation [58]. In addition, besides direct action of LPS on PDAC cells [232], elevated LPS levels during obesity can also induce a shift of intrapancreatic resident macrophages and/or recruited monocytes into pro-inflammatory M1-like macrophages, which may promote PanIN development [80].

## 5. Conclusions

**Figure 2:**
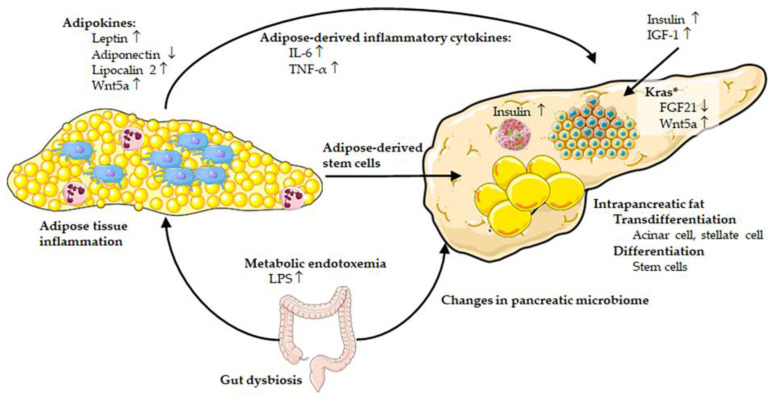
Schematic overview of the mechanisms linking obesity, adipose tissue, and pancreatic cancer as discussed in the main text. During obesity, inflamed (visceral) adipose tissue (adipocytes and resident/recruited immune cells) releases a variety of adipokines (e.g., increase in leptin, lipocalin 2, Wnt5a, and decrease in adiponectin) and adipose-derived inflammatory cytokines (e.g., IL-6 and TNF-α) that may promote proliferation of transformed pancreatic epithelial cells. Obesity-associated gut dysbiosis may lead to metabolic endotoxemia (elevated LPS) that plays a role in adipose tissue inflammation and has direct effects on pancreatic cells. Obesity-associated changes of the gut microbiome may also induce or alter the pancreatic microbiome, which promotes cancer growth. Obesity-associated systemic hyperinsulinemia (and elevated IGF-1) as well as elevated intrapancreatic insulin levels (from pancreatic β-cells) can act as potent growth stimulatory factors for transformed (pre-)neoplastic pancreatic cells. Intrapancreatic adipocytes, either through differentiation of adipose-derived stem cells and/or transdifferentiation of acinar (or pancreatic stellate) cells, may also have a robust impact on pancreatic cancer cell proliferation and changes of the tumor microenvironment. Downstream of oncogenic Kras, the decrease in FGF21 (in transformed pancreatic cells) may render the pancreas susceptible to the pro-tumorigenic effects of obesity. Blue and purple cells within the adipose tissue illustrate various immune cells (e.g., neutrophils (purple) and macrophages (blue)). Yellow circles within the adipose tissue represent adipocytes. Partly created with Servier Medical Art.

Human and preclinical mouse studies have convincingly demonstrated that obesity increases the risk of developing PDAC and promotes PDAC growth. Several mechanisms are generally discussed that underlie the obesity–PDAC connection. A central role is clearly played by adipose tissue and obesity-associated adipose tissue inflammation (Figure 2). 

Soluble adipokines and other inflammatory mediators secreted by the adipose tissue (visceral and/or intrapancreatic) can reach the pancreas systemically and/or via paracrine mechanisms. These mediators can affect metabolism and growth of transformed pancreatic epithelial cells and shape the pancreatic microenvironment. Furthermore, neoplastic pancreatic cells themselves generate pro-tumorigenic factors downstream of oncogenic Kras, which may be augmented and positively reinforced by systemic and paracrine effects of the adipose tissue. Obesity-associated adipose tissue inflammation also plays a major role in creating insulin resistance with ensuing hyperinsulinemia. Elevated insulin levels, systemically and locally, are known as potent growth stimulating factors for transformed pancreatic epithelial cells. Obesity-associated gut dysbiosis may lead to the perturbation of the pancreatic microbiome, which may induce and exacerbate pancreatic inflammation and neoplastic development. In addition, gut dysbiosis as seen in obesity also leads to an impaired gut barrier function and subsequent metabolic endotoxemia, which is thought to be a critical factor in inducing adipose tissue inflammation. Given the central role of adipose tissue in linking obesity with PDAC risk and growth, strategies to target the adipose tissue seem to be of paramount importance to curtail the PDAC promoting actions of obesity. This may be achieved by interventions aimed at inhibiting or reducing obesity-associated adipose tissue inflammation in general or by targeting specific factors that mechanistically initiate and sustain the link between adipose tissue and pancreatic neoplastic cells.
